# Hypoxia‐induced oxidative stress promotes therapy resistance via upregulation of heme oxygenase‐1 in multiple myeloma

**DOI:** 10.1002/cam4.5679

**Published:** 2023-02-12

**Authors:** Ko Abe, Sho Ikeda, Miho Nara, Akihiro Kitadate, Hiroyuki Tagawa, Naoto Takahashi

**Affiliations:** ^1^ Department of Hematology Nephrology, and Rheumatology Akita University Graduate School of Medicine Akita Japan

**Keywords:** heme oxygenase‐1, hypoxia, multiple myeloma, reactive oxygen species, side population

## Abstract

**Background:**

Multiple myeloma (MM) is a hematopoietic malignancy for which proteasome inhibitors have become available in recent years. However, many patients develop resistance to these drugs during treatment. Therefore, it is important to elucidate the mechanisms underlying resistance acquisition by proteasome inhibitors. Side population (SP) cells, which have a high drug efflux capacity and hypoxic responses in the microenvironment have both provided important insights into drug resistance in MM; however, little is known about the characteristics of SP cells in hypoxic microenvironments.

**Methods:**

We performed cDNA microarray analysis for SP and non‐SP obtained from RPMI‐8226 and KMS‐11 cell lines cultured for 48 h in normoxic and hypoxic conditions (1% O_2_). Genes specifically upregulated in hypoxic SP were examined.

**Results:**

Our comprehensive gene expression analysis identified *HMOX1*, *BACH2*, and *DUX4* as protein‐coding genes that are specifically highly expressed in SP cells under hypoxic conditions. We have shown that *HMOX1*/heme oxygenase‐1 (HMOX1/HO‐1) is induced by hypoxia‐inducible reactive oxygen species (ROS) and reduces ROS levels. Furthermore, we found that HMOX1 contributes to hypoxia‐induced resistance to proteasome inhibitors in vitro and in vivo. Excessive ROS levels synergistically enhance bortezomib sensitivity. In clinical datasets, *HMOX1* had a strong and significantly positive correlation with *MAFB* but not *MAF*. Interestingly, hypoxic stimulation increased *MAFB*/*MafB* expression in myeloma cells; in addition, the knockdown of MAFB under hypoxic conditions suppressed HMOX1 expression.

**Conclusion:**

These results suggest that the hypoxia‐ROS‐HMOX1 axis and hypoxia‐induced MafB may be important mechanisms of proteasome inhibitor resistance in hypoxic microenvironments.

## Graphical abstract

By examining in detail the function of genes that are highly expressed in side population (SP) cells exposed to hypoxia, we clarified the contribution of the hypoxia‐ROS‐HMOX1 axis to proteasome inhibitor resistance in hypoxic environments. This axis might also be involved in hypoxia‐induced MafB expression.

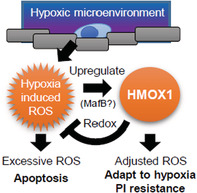

## 1 INTRODUCTION

Multiple myeloma (MM) is a hematopoietic malignancy with a plasma cell phenotype that has seen dramatic advances in treatment in recent years; however, most patients relapse despite prolonged remission.^1^ Although the existence of “myeloma initiating cells” or “myeloma stem‐like cells,” which are the treatment‐resistant fraction responsible for relapse, has been suggested, their phenotype has not yet been identified.^2^ Characterization of these fractions may provide new therapeutic strategies. Recently, several studies on side population (SP) cells and hypoxic responses have been reported as approaches to elucidate the potential drug resistance of myeloma stem‐like cells.

The SP, which is a fraction detected using Hoechst 33342, has higher stemness than the major population (MP). SP cells have been commonly used in studies of cancer stem cells, including solid tumors and hematopoietic tumors, as well as stem cells in normal tissues.^3^, ^4^ In myeloma, it was reported that SP cells are highly tumorigenic but could be suppressed by cerebrone modulators.^5^ We reported high expression of oncogenes such as *AURKA*, *BMI1*, *MYC*, and *IRF4* in myeloma SP cells and the effect of proteasome inhibitors for the fraction.^6^ As myeloma SP is considered a fraction with a large amount of activated stem cell‐like fractions in these reports, factors highly expressed in this fraction may be promising therapeutic targets.

The hypoxic response upregulates various hypoxia‐inducible factors (HIF) target genes, such as *EPO*, to adapt to hypoxia, a state in which insufficient amounts of oxygen are obtained at the tissue level to maintain optimal homeostasis.^7^, ^8^, ^9^ Hypoxic responses in cancers cause therapeutic resistance through various pathways involving neovascularization, noncoding RNAs, and the glycolytic system.^10^, ^11^ In MM, for example, hypoxia leads to undifferentiated phenotypes that may be involved in therapy resistance.^12^, ^13^, ^14^ We have also identified the involvement of hypoxia‐inducible histone‐modifying enzymes and glycolytic enzymes in resistance to therapy.^15^, ^16^, ^17^

Reports on the behavior of the SP cells under hypoxic conditions are very limited. Wen et al. suggested that external stimulation, such as hypoxic stress, can maintain a balance between SP and MP fractions via the activated TGF‐β pathway.^18^ However, there is still a lack of knowledge regarding its role in hypoxic microenvironments, contribution to drug resistance, and therapeutic applications.

In this study, we performed a comprehensive gene expression analysis of hypoxic SP in MM cells and found a possible contribution of oxidative stress‐inducible *HMOX1*/heme oxygenase‐1 to therapeutic resistance.

## 2 MATERIALS AND METHODS

### 2.1 Primary MM samples

This study included five cases of primary MM from Akita University Hospital. Samples were collected according to a protocol approved by the Institutional Review Board of Akita University (No. 1313). Written informed consent was acquired from the study participants before the collection of specimens. The study was conducted with the approval of the Institutional Review Board and according to the Declaration of Helsinki.

### 2.2 Cell lines and cultures

We used four well‐known MM cell lines with various molecular subtypes: RPMI‐8226, KMS‐12‐BM, KMS‐11, and MM.1 S. These cell lines were purchased from the American Type Culture Collection (ATCC). SACHI and SK‐MM‐1 were kindly provided by Dr. Ichiro Hanamura (Aichi Medical University, Aichi, Japan). These cell lines were cultured in RPMI 1640 medium (Thermo Fisher Scientific) supplemented with 10% inactivated fetal calf serum (FCS). 293FT cells were cultured in DMEM containing 10% inactivated fetal calf serum. A multi‐gas incubator MCO‐5 M‐PJ (PHC, Tokyo, Japan) was used for hypoxic culture (1% O_2_).

### 2.3 Analyze and sorting of SP and MP cells

MM cells were resuspended at a concentration of 1 × 10^6^ cells/mL in RPMI 1640 medium containing 10% FCS and 5 μg/mL Hoechst 33342 dye and incubated for 1 h at 37°C. As a negative control, MM cells were preincubated with 100 μmol/L verapamil. Hoechst 33342 dye was used for ultraviolet excitation. SP and MP cells were analyzed and sorted using MoFlo (Beckman Coulter).

### 2.4 cDNA microarray

Gene expression was analyzed using a G2600A SureScan Microarray Scanner System (Agilent). The experimental protocol was performed according to the Agilent Protocol Ver. 6.7. Data were analyzed using GeneSpring (Agilent) and uploaded to GSE207585 in the Gene Expression Omnibus.

### 2.5 Quantitative reverse transcription‐PCR analysis

Total RNA was extracted using TRIzol (Life Technologies). Reverse transcription was performed using the Transcriptor First Strand cDNA Synthesis Kit (Roche). TaqMan probes for GAPDH (Hs02758991_g1), HMOX1 (Hs01110250_m1), and MAFB (Hs00534343_s1) were purchased from Applied Biosystems. Quantitative reverse transcription‐PCR (RT‐qPCR) was performed using Light Cycler 96 (Roche).

### 2.6 Western blot analysis

We used PowerPac Basic, the Mini‐PROTEAN Tetra System, and TransBlot Turbo (Bio‐Rad) for western blot analysis, according to the manufacturer's protocol. HMOX1 (#5853) and MAFB (#30919) antibodies were purchased from Cell Signaling Technology . Tubulin (MS‐581‐P0) was purchased from NeoMarkers.

### 2.7 Transient siRNA transfection

We purchased the following Silencer Select siRNAs from Thermo Fisher Scientific: siHMOX1 #1 (s194530), siHMOX1 #2 (s6673), siMAFB (s19279), siHIF1A #1 (s6539), and siHIF1A #2 (s6541). siRNA transfection was performed using the Nucleofector II and Cell Line Nucleofector Kit V (VCA‐1003; Lonza) according to the manufacturers' protocols. The program “G‐015” was used for the RPMI‐8226 and KMS‐11 cell lines.

### 2.8 Stable knockdown constructs and lentivirus infection

The HMOX1 human shRNA plasmid kit (TL312388), including the control plasmid, was purchased from OriGene . The protocol was previously described.^16^, ^17^ Cells were sorted for GFP expression using a FACSMelody instrument (BD Biosciences).

### 2.9 Reactive oxygen species detection on flow cytometry

The reactive oxygen species (ROS)‐ID Total ROS Detection Kit (ENZ‐51011) was purchased from Enzo Life Sciences. ROS detection was performed using FACSCanto or FACSLyric (BD Biosciences), according to the manufacturer's protocol.

### 2.10 Cell viability assay

MTS assays were performed using the CellTiter 96 AQueous One Solution Cell Proliferation Assay (Promega) according to the manufacturer's protocol. Apoptosis assays were using APC‐Annexin V (550474), 7‐AAD (559925), and Annexin V binding buffer (556454) purchased from BD Biosciences and were performed using FACSLyric (BD Biosciences).

### 2.11 Xenograft mouse model

KMS‐11 cells (1 × 10^6^ cells) were subcutaneously injected into the right or left side of the bodies of 6‐ to 8‐week‐old female NOD/Shi‐scid IL‐2γnul (NOG) mice (Central Institute for Experimental Animals, Kawasaki, Japan). The protocols for animal experimentation described in this study were approved by the Animal Committee of Akita University (approval no. a‐1‐0358).

### 2.12 Reagents

Bortezomib (021–18,901) and N‐acetyl‐l‐cysteine (NAC) (017–05131) were purchased from FUJIFILM Wako Pure Chemicals. Phorbol 12‐Myristate 13‐Acetate (P8139) was purchased from Sigma‐Aldrich.

### 2.13 Statistical analysis

Data were analyzed using the Student's *t*‐test, Mann–Whitney *U*‐test, or two‐way ANOVA. Bars represent the mean ± 95% confidence interval (CI) of three independent experiments. Asterisks (*) indicate statistical significance: *0.01 *≤ p* < 0.05; **0.001 *≤ p* < 0.01; ***p < 0.001; NS—not significant.

## 3 RESULTS

### 3.1 SP cell sorting of myeloma cell lines cultured under hypoxic conditions

**FIGURE 1:**
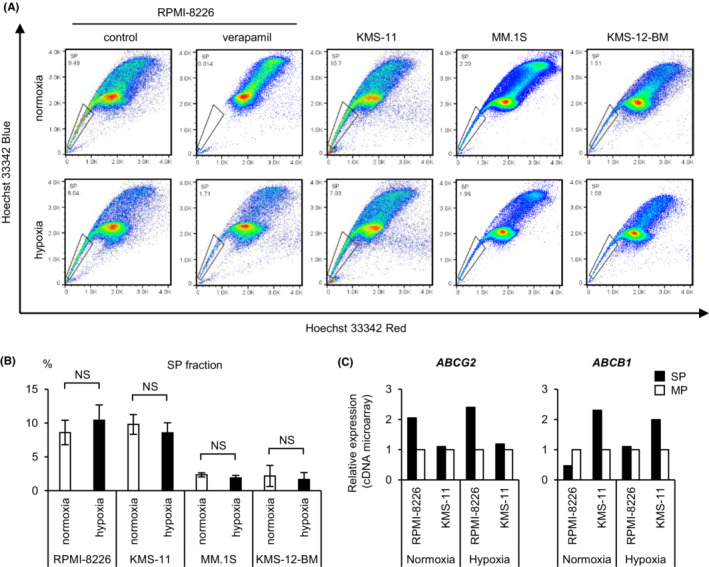
Detection of side population cells under normoxic or hypoxic conditions in myeloma cell lines. (A) The flow cytometry analysis of side population (SP) cells is shown. Myeloma cell lines were incubated under normoxic or hypoxic conditions for 48 h and then incubated in 5 μg/mL Hoechst 33342 alone or with 100 μM verapamil for 1 h. The enclosed sections indicate the SP. X axis: Hoechst 33342 Red; Y axis: Hoechst 33342 Blue. (B) The percentage of SP cells among the indicated myeloma cell lines cultured under normoxic or hypoxic conditions for 48 h. NS—not significant. Bars represent the mean ± 95% CI of three or more replicates. (C) cDNA microarray analysis of RPMI‐8226 and KMS‐11. The expression levels of *ABCG2* and *ABCB1* in SP vs. major population (MP) cells are shown.

First, to perform a comprehensive gene expression analysis of hypoxic SP cells, we detected SP cells in four myeloma cell lines (RPMI‐8226, KMS‐11, MM.1 S, and KMS‐12‐BM) cultured for 48 h under normoxic or hypoxic conditions using flow cytometry (Figure 1A). It has been reported that hypoxic stress increases the expression of *ABCG2* and *ABCB1*, which may be responsible for the drug efflux capacity of SP cells.^19^, ^20^ Thus, we expected the percentage of SP cells to increase under hypoxic stress. Unexpectedly, there was no significant difference in the percentage of SP cells between the hypoxic and normoxic cultures among any of the four myeloma cell lines (Figure 1B). The SP and MP of RPMI‐8226 and KMS‐11 cells were sorted for normoxic and hypoxic cultures, respectively, and cDNA microarray assays were performed on these samples. In RPMI‐8226 and KMS‐11 cells, the expression of *ABCG2* or *ABCB1* in SP cells was higher than that in MP cells (Figure 1C). The expression of *ABCB1* in RPMI‐8226 cells under normoxic conditions was too low to be confirmed by RT‐qPCR (data not shown). The SP of RPMI‐8226 is dependent on *ABCG2* expression rather than *ABCB1*. Altogether, these data showed that SP cells of myeloma cell lines could be reliably sorted under each oxygen condition.

### 3.2 *HMOX1* is specifically highly expressed in hypoxic SP cells

**FIGURE 2:**
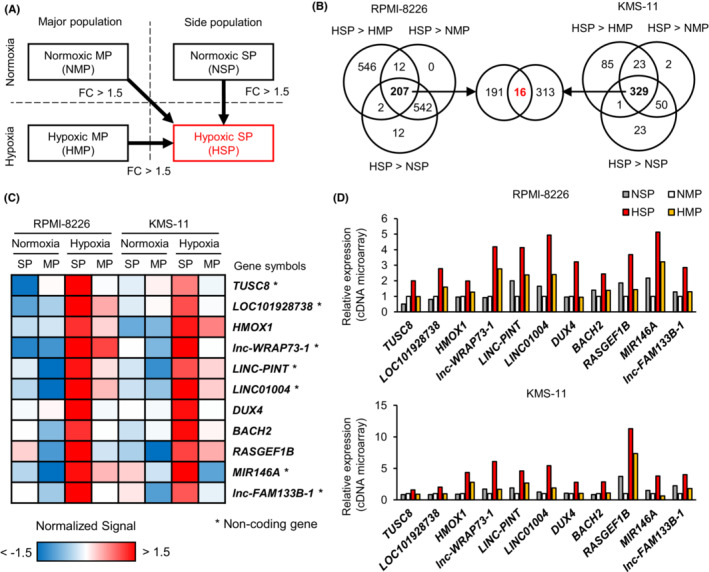
Identification of specifically upregulated genes in hypoxic side population cells in myeloma cells. (A) The method of identifying hypoxic side population (SP)‐specific genes is shown in the schema. FC, fold change. (B) Venn diagram showing the number of probes that met the criteria. There were 16 probes that were determined to be hypoxic SP‐specific in both RPMI‐8226 and KMS‐11. (C) Heat map showing the expression of 11 hypoxic SP‐specific genes identified in Figure 3B. Asterisks (*) indicate noncoding genes. (D) Expression of 11 hypoxic SP‐specific genes. The expression levels of *HMOX1*, *DUX4*, and *BACH2* did not differ between SP and MP cells under normoxic conditions but were greater in SP cells under hypoxic conditions.

To identify functional genes in hypoxic SP cells, we extracted genes whose expression was upregulated by more than 1.5‐fold in hypoxic SP cells compared with that in normoxic MP, normoxic SP, and hypoxic MP cells (Figure 2A). There were 207 hypoxic SP‐specific high‐signal probes that met these criteria in RPMI‐8226 and 329 in KMS‐11, and 16 probes were common between them (Figure 2B). Among these, 11 genes (*TUSC8*, *LOC101928738*, *HMOX1*, *lnc‐WRAP73‐1*, *LINC‐PINT*, *LINC01004*, *DUX4*, *BACH2*, *RASGEF1B*, *MIR146A*, and *lnc‐FAM133B‐1*) with gene symbols were identified (Figure 2C; Table S1). We focused on protein‐coding genes (including *HMOX1*, *DUX4*, and *BACH2*) that showed little difference in intensity between SP and MP cells in normoxia but with clearly higher intensity in SP cells compared with that in MP cells in hypoxia (Figure 2D). However, RT‐qPCR showed very low expression of *BACH2* and no expression of *DUX4*; therefore, these genes were excluded from the analysis. Therefore, we investigated the function of *HMOX1*/heme oxygenase‐1 in hypoxic environments. One of the functions of HMOX1 is to protect cells from apoptosis induced by ROS.^21^ The role of HMOX1 in hypoxic environments is largely unknown in MM. In addition, our microarray data showed that myeloma patient samples exposed to hypoxia had high expression of *HMOX1* but not of the isozyme *HMOX2* (Figure S1). Therefore, we hypothesized that ROS and HMOX1 are involved in promoting therapeutic resistance in hypoxic microenvironments and performed subsequent experiments.

### 3.3 HMOX1 neutralizes hypoxia‐induced ROS

**FIGURE 3:**
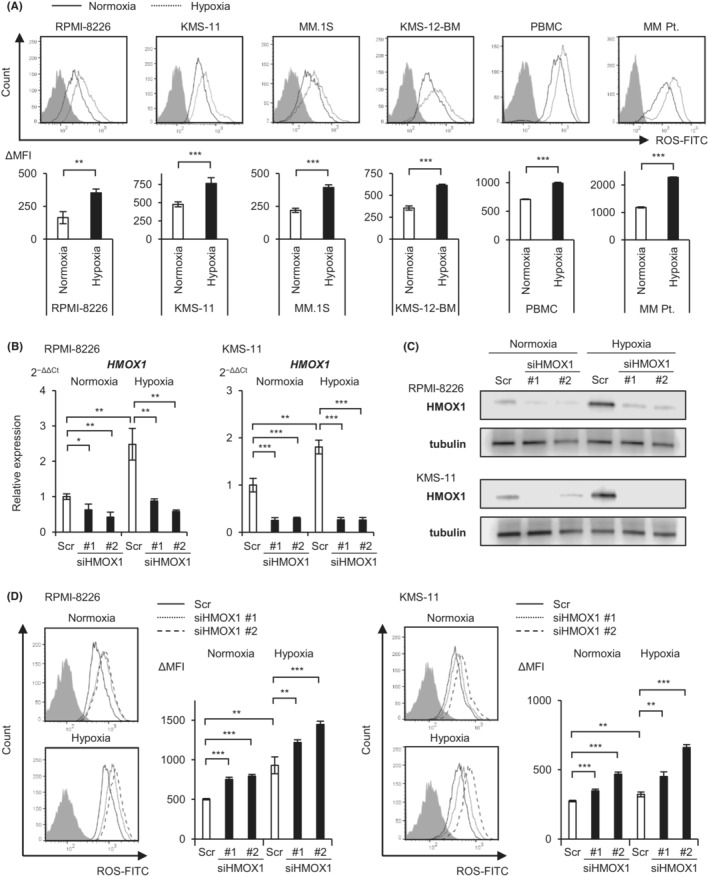
Hypoxic stress‐induced reactive oxygen species are reduced by hypoxia‐inducible heme oxygenase‐1. (A) Flow cytometry of reactive oxygen species (ROS) in myeloma cell lines, one myeloma patient specimen, and normal peripheral blood mononuclear cells (PBMC) cultured for 48 h under normoxic and hypoxic conditions. Solid lines indicate normoxia, and dashed lines indicate hypoxia. The ΔMFI (mean fluorescence intensity) values are shown in the lower panel. (B) qRT‐PCR analysis of *HMOX1* in RPMI‐8226 and KMS‐11 cell lines transiently transduced with siHMOX1 #1, siHMOX1 #2, and control scrambled siRNA (Scr) and cultured under normoxic or hypoxic conditions for 72 h. (C) Western blot analysis of HMOX1 in RPMI‐8226 and KMS‐11 cell lines transiently transduced with siHMOX1 #1, siHMOX1 #2, and control scrambled siRNA (Scr) and cultured under normoxic or hypoxic conditions for 72 h. (D) Flow cytometry of ROS in myeloma cell lines transiently transduced with siHMOX1 #1, siHMOX1 #2, and control scrambled siRNA (Scr) and cultured for 72 h under normoxic and hypoxic conditions. Solid lines indicate Scr, and dashed lines indicate shHMOX1. The ΔMFI values are shown in the right panel. Asterisks (*) indicate statistical significance: *0.01 ≤ *p <* 0.05; **0.001 ≤ *p <* 0.01; *** *p <* 0.001; NS—not significant. The Student's *t*‐test was used to test for significance. Bars represent the mean ± 95% CI of three replicates.

Hypoxic stress induces ROS in various tissues.^22^, ^23^, ^24^ We therefore examined the levels of ROS in normoxic or hypoxic cultures for 48 h by flow cytometry using myeloma cell lines, including RPMI‐8226, KMS‐11, MM.1 S, and KMS‐12‐BM, a myeloma patient sample, and normal mononuclear cells. We found a significant increase in ROS in all cell types under hypoxic conditions compared with normoxic conditions, suggesting that hypoxic stress induces oxidative stress in myeloma cells (Figure 3A). The rate of increase in ROS levels was higher in myeloma cell lines and patient samples than in normal mononuclear cells (approximately 2‐ and 1.4‐fold, respectively). We then transfected two sequences of HMOX1 siRNA and a scrambled control into RPMI‐8226 and KMS‐11 cells by electroporation, incubated them under normoxic and hypoxic conditions for 72 h, and examined *HMOX1* mRNA and HMOX1 protein levels by RT‐qPCR and western blotting, respectively. The results confirmed that hypoxia increased *HMOX1*/HMOX1 expression and that knockdown effects of siRNAs occurred under both oxygen conditions (Figure 3B,C). We used these siRNAs to observe their effects on ROS levels. The same approach was used to transiently introduce siHMOX1 into KMS‐11 and RPMI‐8226 cells: these were incubated under normoxic and hypoxic conditions for 72 h, and ROS levels were then measured. Flow cytometry revealed that HMOX1 knockdown significantly increased ROS levels under both normoxic and hypoxic conditions (Figure 3D). These results suggest that hypoxic stress induces ROS and that HMOX1 can neutralize hypoxia‐induced ROS.

### 3.4 ROS, but not HIF, upregulates HMOX1 expression under hypoxic conditions in MM cells

**FIGURE 4:**
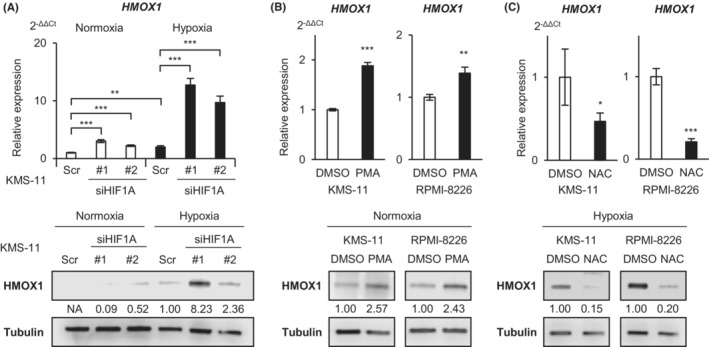
Heme oxygenase‐1 is upregulated via oxidative stress but not hypoxia‐inducible factor‐1 under hypoxic conditions. (A) qRT‐PCR analysis (upper panel) and western blot analysis (lower panel) of *HMOX1*/HMOX1 in the KMS‐11 cell line transiently transduced with siHIF1 #1, siHIF1 #2, and control scrambled siRNA (Scr) and cultured under normoxic or hypoxic conditions for 72 h. (B) qRT‐PCR analysis (upper panel) and western blot analysis (lower panel) of *HMOX1*/HMOX1 in KMS‐11 and RPMI‐8226 cell lines cultured with PMA or vehicle (DMSO) under normoxic conditions for 24 h. (C) qRT‐PCR analysis (upper panel) and western blot analysis (lower panel) of *HMOX1*/HMOX1 in KMS‐11 and RPMI‐8226 cell lines cultured under hypoxic conditions for 48 h and then cultured with N‐acetyl‐l‐cysteine or vehicle (DMSO) under hypoxic conditions for 24 h. Asterisks (*) indicate statistical significance: *0.01 ≤ *p <* 0.05; **0.001 ≤ *p <* 0.01; *** *p <* 0.001; the Student's *t*‐test was used to test for significance. Bars represent the mean ± 95% CI of three replicates.

We investigated the regulatory mechanisms of HMOX1 under hypoxic conditions. It is well‐known that many hypoxia‐inducible genes have HIF‐binding elements and are positively regulated by the transcriptional activity of HIF.^25^ In our previous study, the increased expression of hypoxia‐inducible genes such as *HK2*, *SLC2A1*, and *KDM3A* was canceled by HIF knockdown under hypoxic conditions.^16^, ^17^ Unexpectedly, the RT‐qPCR and western blot analyses showed that *HMOX1*/HMOX1 expression was not decreased by HIF knockdown, rather it was significantly increased (Figure 4A). It has been reported that ROS induces HMOX1 in leukemia cells.^26^ The addition of phorbol 12‐myristate 13‐acetate (PMA), a ROS inducer, significantly increased *HMOX1*/HMOX1 in myeloma cells (Figure 4B). Conversely, the addition of NAC, a ROS neutralizer, suppressed *HMOX1*/HMOX1 expression under hypoxic conditions (Figure 4C). These results suggest that HMOX1 is induced in hypoxic environments by a ROS‐mediated pathway rather than HIF in myeloma cells.

### 3.5 Knockdown of HMOX1 attenuates hypoxia‐induced proteasome inhibitor resistance

**FIGURE 5:**
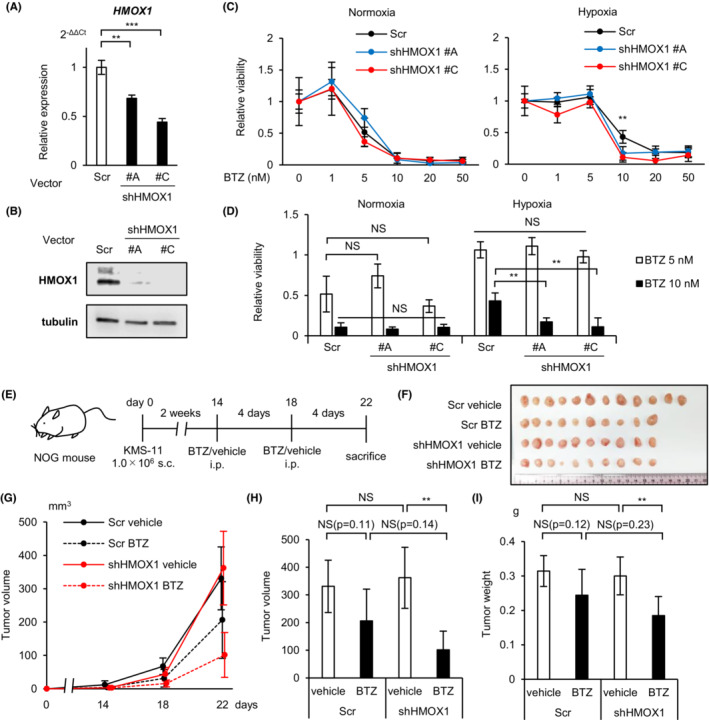
Stable knockdown of HMOX1 enhanced the antimyeloma effect of bortezomib *in vitro* and *in vivo*. (A) qRT‐PCR analysis of HMOX1 in KMS‐11 cells stably transduced with shHMOX1 #A, #C, and control scrambled shRNA (Scr). (B) Western blot analysis of HMOX1 in KMS‐11 cells stably transduced with shHMOX1 #A, #C, and control scrambled shRNA (Scr). (C) MTS assay for KMS‐11 stably transduced with shHMOX1 #A, #C, and control scrambled shRNA (Scr) cultured under normoxic or hypoxic conditions with indicated concentrations of bortezomib (BTZ) for 48 h. (D) A graph showing a sampling of the results of two bortezomib concentrations (5 nM and 10 nM) in the MTS assay is shown. (E) Illustration of the protocol of the in vivo transplantation and treatment. 1 × 10^6^ of shHMOX1 #C or control shRNA stably‐transduced KMS‐11 cells were inoculated into NOG mice. Mice were treated with bortezomib (1.0 mg/kg) or phosphate‐buffered saline intraperitoneally. Scr‐vehicle; *n* = 12, Scr‐BTZ; *n* = 10, shHMOX1‐vehicle; *n* = 10, and shHMOX1‐BTZ; *n* = 10. BTZ—bortezomib. s.c.—subcutaneous. i.p.—intraperitoneal. (F) The tumor photographs from each group are shown. (G) The tumor growth curves of each group are shown. X axis, days after transplantation (days); Y axis, tumor volume (mm^3^, major×minor^2^/2). (H) The tumor volume at day 22 of each group is shown. (I) The tumor weight at the time the mice were sacrificed of each group is shown. Asterisks (*) indicate statistical significance: **0.001 ≤ *p <* 0.01; *** *p <* 0.001; NS—not significant. The Student's *t*‐test was used to test for significance. Bars represent the mean ± 95% CI of three replicates.

To investigate the long‐term effects of HMOX1 knockdown, vectors containing different shRNAs (#A‐D) against HMOX1 and control shRNA were introduced into KMS‐11 and sorted using GFP to establish stable knockdown cell lines. We confirmed the knockdown efficiency by RT‐qPCR and western blotting and found that the knockdown efficiency of #A and #C was favorable (Figure 5A,B). Next, we examined the phenotypes of the knockdown cell lines. No significant difference was found in the apoptosis percentage or growth curves between control and shHMOX1 transductions under both normoxic and hypoxic conditions (Figure S2).

The effect of proteasome inhibitors has been reported to diminish in hypoxic environments.^16^, ^27^ The reason for this is not fully understood. Proteasome inhibitors have been reported to induce apoptosis in myeloma cells not only through proteasome inhibition but also through the production of ROS.^28^ Therefore, we investigated the involvement of HMOX1 in the effects of bortezomib in hypoxic environments. MTS assays showed that bortezomib against HMOX1 knockdown KMS‐11 reduced cell viability under hypoxic rather than normoxic conditions (Figure 5C,D). We further investigated the effects of HMOX1 knockdown on the effects of bortezomib in vivo. We transplanted HMOX1 knockdown or control KMS‐11 into immunodeficient mice (NOG mice), injected bortezomib (1.0 mg/kg) intraperitoneally on days 14 and 18, and sacrificed the mice on day 22 (Figure 5E). There was no significant difference in subcutaneous tumor volume and weight between the control and HMOX1 knockdown cell lines, whereas HMOX1 knockdown resulted in a significant bortezomib‐induced reduction in subcutaneous tumor volume and weight (Figure 5F–I). In the control and HMOX1 knockdown groups, bortezomib reduced tumor diameter by 37.7% and 71.9% (fold change 1.91), respectively, and decreased tumor weight by 22.3% and 38.8% (fold change 1.72), respectively. It has been shown that hypoxia is important for the formation of extramedullary lesions and plasmacytomas.^29^, ^30^ Therefore, these in vitro and in vivo results suggest that hypoxia‐inducible HMOX1 may partially contribute to the induction of bortezomib resistance in hypoxic microenvironments.

### 3.6 Excessive ROS levels enhance the effect of bortezomib

**FIGURE 6:**
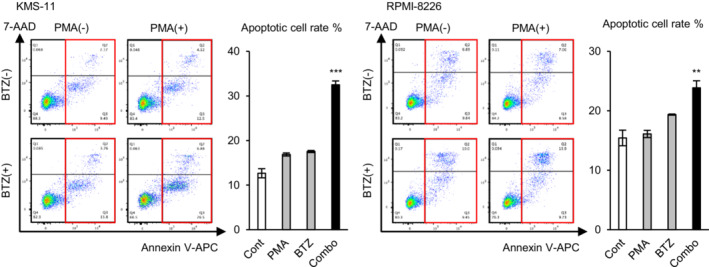
Reactive oxygen species and proteasome inhibitors synergistically induce apoptosis in myeloma cells. Apoptosis assay of KMS‐11 and RPMI‐8226 cells cultured with 5 nM bortezomib (BTZ) and/or 100 ng/mL PMA. Asterisks (*) indicate statistical significance: ** 0.001 ≤ *p <* 0.01; *** *p <* 0.001. The two‐way ANOVA was used to test for the significance of the interaction. Bars represent the mean ± 95% CI of three replicates.

As the experiments performed above suggested that the ROS‐neutralizing effect of HMOX1 induces bortezomib resistance, we examined whether excessive levels of ROS enhance the effect of bortezomib. PMA and a low concentration of bortezomib (5 nM, which is lower than that for IC50) were added to KMS‐11 and RPMI‐8226, and an apoptosis assay was performed. As a result, PMA or a low concentration of bortezomib increased apoptotic cells by only a small percentage, while simultaneous exposure to both significantly increased apoptotic cells synergistically in two cell lines (Figure 6). This result indicates that excessive ROS levels enhance the antimyeloma effect of proteasome inhibitors.

### 3.7 *HMOX1* and *MAFB* expression are positively correlated

**FIGURE 7:**
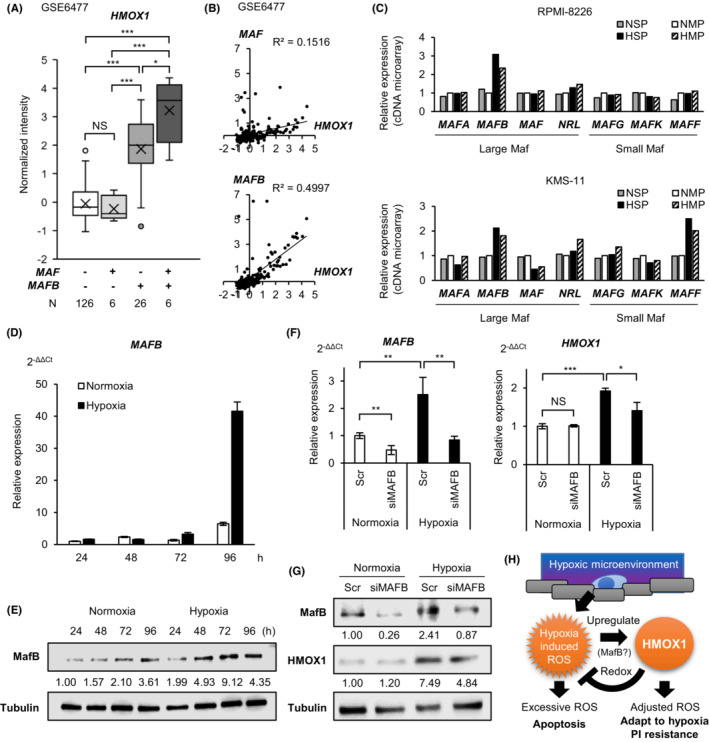
*MAFB* is positively correlated with *HMOX1* and may upregulate *HMOX1*/HMOX1 under hypoxic conditions in myeloma cells. (A) The expression of *HMOX1* in four groups classified by *MAF* and *MAFB* expression in primary MM cases. (B) The correlations between *MAF* and *HMOX1* (upper panel) and *MAFB* and *HMOX1* (lower panel). (C) Expression of large Maf (*MAFA*, *MAFB*, *MAF*, and *NRL*) and small Maf (*MAFG*, *MAFK*, and *MAFF*) in normoxic side population (NSP), normoxic MP (NMP), hypoxic SP (HSP), and hypoxic MP (HMP) RPMI‐8226 and KMS‐11 cells. (D) qRT‐PCR analysis of *MAFB* in the KMS‐11 cell line cultured under normoxic or hypoxic conditions for 24, 48, 72, and 96 h. (E) Western blot analysis of MafB in the KMS‐11 cell line cultured under normoxic or hypoxic conditions for 24, 48, 72, and 96 h. (F) qRT‐PCR analysis of *MAFB* and *HMOX1* in the KMS‐11 cell line transiently transduced with siMAFB and control scrambled siRNA (Scr) and cultured under normoxic or hypoxic conditions for 72 h. (G) Western blot analysis of MafB and HMOX1 in the KMS‐11 cell line transiently transduced with siMAFB and control scrambled siRNA (Scr) and cultured under normoxic or hypoxic conditions for 72 h. (H) Schematic illustration of the role of hypoxia‐induced ROS, HMOX1, and MafB in proteasome inhibition in a hypoxic environment. Asterisks (*) indicate statistical significance: *0.01 ≤ *p <* 0.05; **0.001 ≤ *p <* 0.01; *** *p <* 0.001; NS—not significant. The Mann–Whitney *U* test (for Figure 7A) or Student's *t*‐test (for Figure 7F) was used to test for significance. Bars represent the mean ± 95% CI of three replicates.

To further elucidate the regulatory mechanisms of *HMOX1* in the clinical setting, we first examined whether *HMOX1* expression differs among MM subtypes using the published dataset GSE4581 in silico. The results showed that HMOX1 expression was higher in the cluster characterized by higher large Maf (mainly *MAF* and *MAFB* in MM) expression compared with that in the other clusters (Figure S3A). We examined *HMOX1* expression in the *MAF* alone high‐expressing group, *MAFB* alone high‐expressing group, both high‐expressing groups, and both low‐expressing groups using another dataset, GSE6477 (Figure S3B). Interestingly, we found that *HMOX1* expression was significantly higher in the *MAFB* high‐expressing group and both high‐expressing groups than in both low‐expressing groups, whereas there was no significant difference in *HMOX1* expression in the *MAF*‐only high‐expressing group compared with both low‐expressing groups (Figure 7A). Furthermore, the positive correlation between *MAFB* and *HMOX1* was stronger than that between *MAF* and *HMOX1* (Figure 7B). *MAFB* is overexpressed by t(14;20)(q32;q12) in MM cells and is associated with a poor prognosis.^31^, ^32^ However, in this dataset, similar to actual clinical practice, only a few cases may have a high expression of *MAF* or *MAFB* due to translocation. We examined the HMOX1 expression in myeloma cell lines, harboring t(14;20), such as SACHI and SK‐MM‐1. HMOX1 expression was independent of the presence of t(14;20) (Figure S3C). This result suggests that HMOX1 was likely associated with MafB, induced epigenetically by external stimulation, rather than translocation. Our microarray data showed that *MAFB* was highly expressed in hypoxic SP (Figure 7C; Table S2).

We investigated whether MafB regulates *HMOX1*/HMOX1 under hypoxic conditions. We found that *MAFB*/MafB was induced by hypoxia even in a cell line without t(14;20), such as KMS‐11 (Figure 7D,E). MAFB knockdown was performed on KMS‐11 cells, and *HMOX1*/HMOX1 expression was examined under normoxic and hypoxic conditions. The results showed that the knockdown of MAFB in normoxia resulted in no significant difference in *HMOX1*/HMOX1 expression, whereas the knockdown of MAFB in hypoxia significantly decreased *HMOX1*/HMOX1 expression (Figure 7F,G). These results suggest that MafB induction is involved in the elevated expression of HMOX1 under hypoxic conditions. Together, MafB is implicated in the hypoxia‐ROS‐HMOX1 axis that contributes to proteasome inhibitor resistance (Figure 7H).

## 4 DISCUSSION

In this study, we examined in detail the function of genes that are highly expressed in SP cells exposed to hypoxia and clarified a new role for HMOX1 in contributing to proteasome inhibitor resistance. Suitable levels of HMOX1 in cancer cells have been reported to exert cytoprotective effects via their antioxidant effects.^33^ The involvement of HMOX1 in myeloma cells has been reported in several studies. Importantly, HMOX1 expression is increased by bortezomib.^34^ It has also been reported that HMOX1 induces bortezomib resistance by inhibiting the APRIL‐NF‐κB‐CCL3 signaling pathways and interfering with the ERK‐STAT3‐Gas6 axis.^35^, ^36^ These reports suggest that HMOX1 could affect the antimyeloma effect of bortezomib in a negative feedback manner. However, although HMOX1 expression is induced by hypoxia, the critical function of HMOX1 in myeloma cells adapted to a hypoxic microenvironment remains unclear. The antimyeloma effect of proteasome inhibitors is thought to be mainly due to increased endoplasmic reticulum stress; however, excessive ROS production is also believed to be involved.^37^ Indeed, HDAC inhibitors that strongly induce ROS have been used in clinical practice in combination with bortezomib.^38^, ^39^ In addition, a certain HDAC4/5 selective inhibitor induces apoptosis via a decrease in HMOX1 expression.^40^ Notably, HMOX1 is also involved in lenalidomide resistance.^41^ These reports suggest that HMOX1, which can reduce ROS, has a significant impact on the efficacy of antimyeloma drugs. Thus, a better understanding of the roles of HMOX1 and ROS in hypoxic microenvironments is required.

It is known that mitochondria‐derived ROS paradoxically increase in hypoxic environments.^22^, ^23^, ^24^ These hypoxia‐induced ROS may be deeply involved in the pathogenesis of cancer. For example, in hypovascular pancreatic cancer, hypoxia‐inducible ROS promotes cell survival via autophagic degradation of aberrant mucin.^42^ Furthermore, hypoxia‐inducible ROS have been shown to induce chemotherapy resistance in several solid tumors.^43^, ^44^ However, the functions of hypoxia‐inducible ROS in myeloma have not been studied in detail. Here, we showed that ROS were induced in myeloma cells more strongly than in normal cells after hypoxia exposure. Furthermore, our results show that hypoxia‐inducible ROS induces HMOX1, which contributes to bortezomib resistance in hypoxic environments. As HMOX1 can reduce ROS, providing excessive ROS may be a possible therapeutic strategy for adapting myeloma cells to hypoxia. Our data suggest that excessive ROS levels can synergistically enhance the effects of proteasome inhibitors in myeloma cells.

It is also known that ROS production under hypoxic conditions is a major non‐HIF‐dependent pathway for adaptation to hypoxia.^22^, ^23^, ^24^ It is generally believed that *HMOX1* is regulated by HIF.^45^ Indeed, it was recently reported that HIF‐regulated *HMOX1* contributes to the maintenance of the undifferentiated phenotype in the SP of Hodgkin lymphoma.^46^ However, our data suggest that ROS, not HIF, is responsible for the elevated expression of HMOX1 in hypoxic environments. Gene expression for cell survival under hypoxic environments may require not only precise regulation of HIF levels but also precise regulation of ROS levels. Thus, interference with ROS levels may have a critical impact on cellular homeostasis in myeloma cells adapted to hypoxic microenvironments. This could be an important treatment strategy for MM in the future.

We found that even in the absence of translocation, hypoxic stress increased the expression of *MAFB*/MafB, which is a poor prognostic factor in myeloma,^47^ and that *MAFB* may be involved in *HMOX1* expression. Various downstream genes may be involved in the therapeutic resistance caused by MafB (a transcription factor) overexpression. In this study, we found that the target of inducible MafB in the microenvironment might be *HMOX1*. A past study showed that *CCND2*, *CCR1*, and *ITGB7* are known targets of c‐Maf, which belong to the same large Maf group as MafB.^48^ On the contrary, knowledge of MafB‐specific targets is insufficient. Previously, genes regulated by MafB have been comprehensively reviewed.^49^ In the supplementary data for this report, *HMOX1* was included in the list of genes that could be affected by MafB expression; however, its significance has not been discussed.^49^ As the present study was not able to comprehensively analyze the transcriptional target genes of hypoxia‐induced MafB or examine the actual binding of MafB, further study of the role of MafB in hypoxia is necessary for the future. As it has been suggested that MafB and HMOX1 induce bortezomib resistance,^50^ it can be assumed that inhibiting oxidative stress reduction mechanisms is another possible way to cancel bortezomib resistance.

In summary, we clarified the contribution of the hypoxia‐ROS‐HMOX1 axis to proteasome inhibitor resistance in hypoxic environments. This axis might also be involved in hypoxia‐induced MafB expression. Because mechanisms that reduce excessive ROS in hypoxic microenvironments are thought to be closely related to cell survival and drug resistance, targeting this mechanism may be a new strategy to overcome drug resistance in refractory MM.

## AUTHOR CONTRIBUTIONS

**Ko Abe:** Formal analysis (equal); investigation (equal); writing – original draft (equal); writing – review and editing (equal). **Sho Ikeda:** Conceptualization (lead); data curation (lead); formal analysis (lead); funding acquisition (equal); investigation (equal); methodology (lead); project administration (lead); resources (equal); writing – original draft (equal); writing – review and editing (equal). **Miho Nara:** Conceptualization (equal); funding acquisition (equal); methodology (equal); resources (equal). **Akihiro Kitadate:** Data curation (equal); formal analysis (equal); validation (equal). **Hiroyuki Tagawa:** Conceptualization (equal); funding acquisition (equal); methodology (equal); supervision (equal). **Naoto Takahashi:** Data curation (equal); resources (equal); software (equal); supervision (equal).

## CONFLICT OF INTEREST STATEMENT

NT received honoraria from Pfizer, Otsuka, and Novartis, research funds from Novartis and Otsuka, and scholarship from Eisai, Otsuka, Asahi‐Kasei, and Ono. SI and AK received honoraria from Janssen.

## Supporting information


Data S1:

## Data Availability

Data were analyzed using GeneSpring (Agilent) and uploaded to GSE207585 in the Gene Expression Omnibus.
