# Supplementary material for: Hypoxia‐induced oxidative stress promotes therapy resistance via upregulation of heme oxygenase‐1 in multiple myeloma
Source: Cancer Med. 2023 Feb 12;12(8):9709–22. doi: 10.1002/cam4.5679 (PMC10166934; doi:10.1002/cam4.5679)
Supplement: Supplementary file 1 — Data S1: [file CAM4-12-9709-s001.pdf]

Table S1. Relative expression of genes upregulated in hypoxic side population cells									
		8226	8226	8226	8226	KMS-11	KMS-11	KMS-11	KMS-11
ProbeName	GeneSymbol	NSP	NMP	HSP	HMP	NSP	NMP	HSP	HMP
A_33_P3546363	*TUSC8*	0.51	1.00	2.00	0.98	0.84	1.00	1.59	0.89
A_19_P00318813	*LOC101928738*	0.81	1.00	2.77	1.59	0.86	1.00	2.02	0.96
A_23_P120883	*HMOX1*	0.96	1.00	1.98	1.28	0.91	1.00	4.35	2.81
A_21_P0001093	*lnc-WRAP73-1*	0.92	1.00	4.18	2.77	1.71	1.00	6.08	1.69
A_19_P00316000	*LINC-PINT*	2.00	1.00	4.14	2.37	1.89	1.00	4.60	2.68
A_33_P3762918	*LINC01004*	1.65	1.00	4.94	2.41	1.26	1.00	5.42	1.89
A_33_P3248602	*DUX4*	0.95	1.00	3.21	0.95	1.04	1.00	2.80	1.03
A_33_P3277714	*BACH2*	1.40	1.00	2.44	1.39	0.84	1.00	2.86	1.09
A_33_P3364646	*RASGEF1B*	1.86	1.00	3.68	1.44	3.73	1.00	11.28	7.36
A_33_P3718269	*MIR146A*	2.18	1.00	5.12	3.22	1.48	1.00	3.79	0.63
A_33_P3307980	*lnc-FAM133B-1*	1.29	1.00	2.85	1.29	2.22	1.00	4.01	1.81

Table S2. Relative expression of large or small Maf genes in side population or major population cells under normoxic or hypoxic conditions

		8226	8226	8226	8226	KMS-11	KMS-11	KMS-11	KMS-11
ProbeName	GeneSymbol	NSP	NMP	HSP	HMP	NSP	NMP	HSP	HMP
A_24_P127719	*MAFA*	0.81	1.00	0.98	1.03	0.87	1.00	0.63	0.97
A_33_P3220837	*MAFB*	1.20	1.00	3.08	2.34	0.94	1.00	2.12	1.80
A_23_P397376	*MAF*	0.99	1.00	0.95	1.12	0.95	1.00	0.45	0.55
A_33_P3423027	*NRL*	0.94	1.00	1.28	1.47	1.06	1.00	1.18	1.66
A_23_P78209	*MAFG*	0.75	1.00	0.89	0.92	0.89	1.00	1.04	1.35
A_23_P373598	*MAFK*	1.02	1.00	0.80	0.75	0.89	1.00	0.71	0.80
A_23_P103110	*MAFF*	0.63	1.00	0.97	1.10	0.99	1.00	2.49	2.01

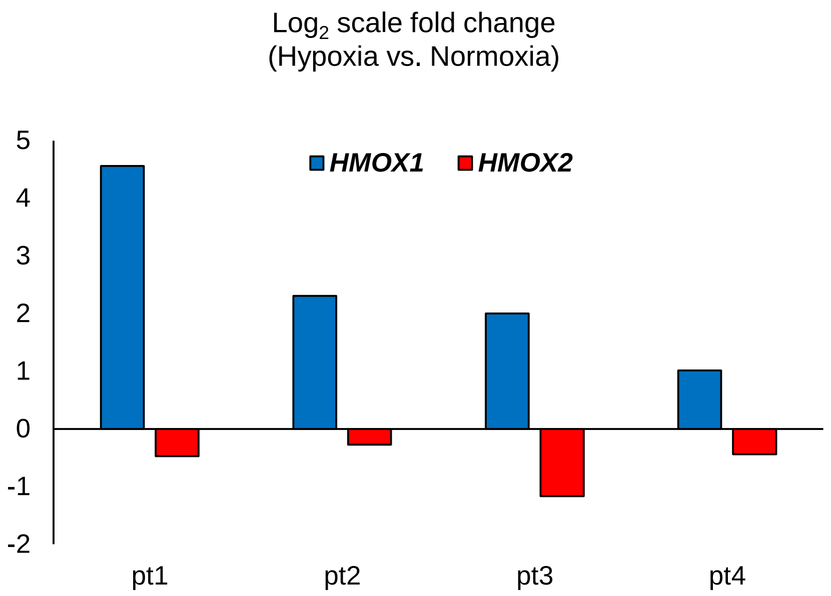

Figure S1. Gene expression change of *HMOX1* and *HMOX2* in patient samples cultured under hypoxic conditions *vs*. normoxic conditions.
Samples (n=4) were cultured under normoxic or hypoxic conditions for 48 h. Hypoxia increased *HMOX1* expression but decreased *HMOX2* expression. The data were uploaded to GSE80545.

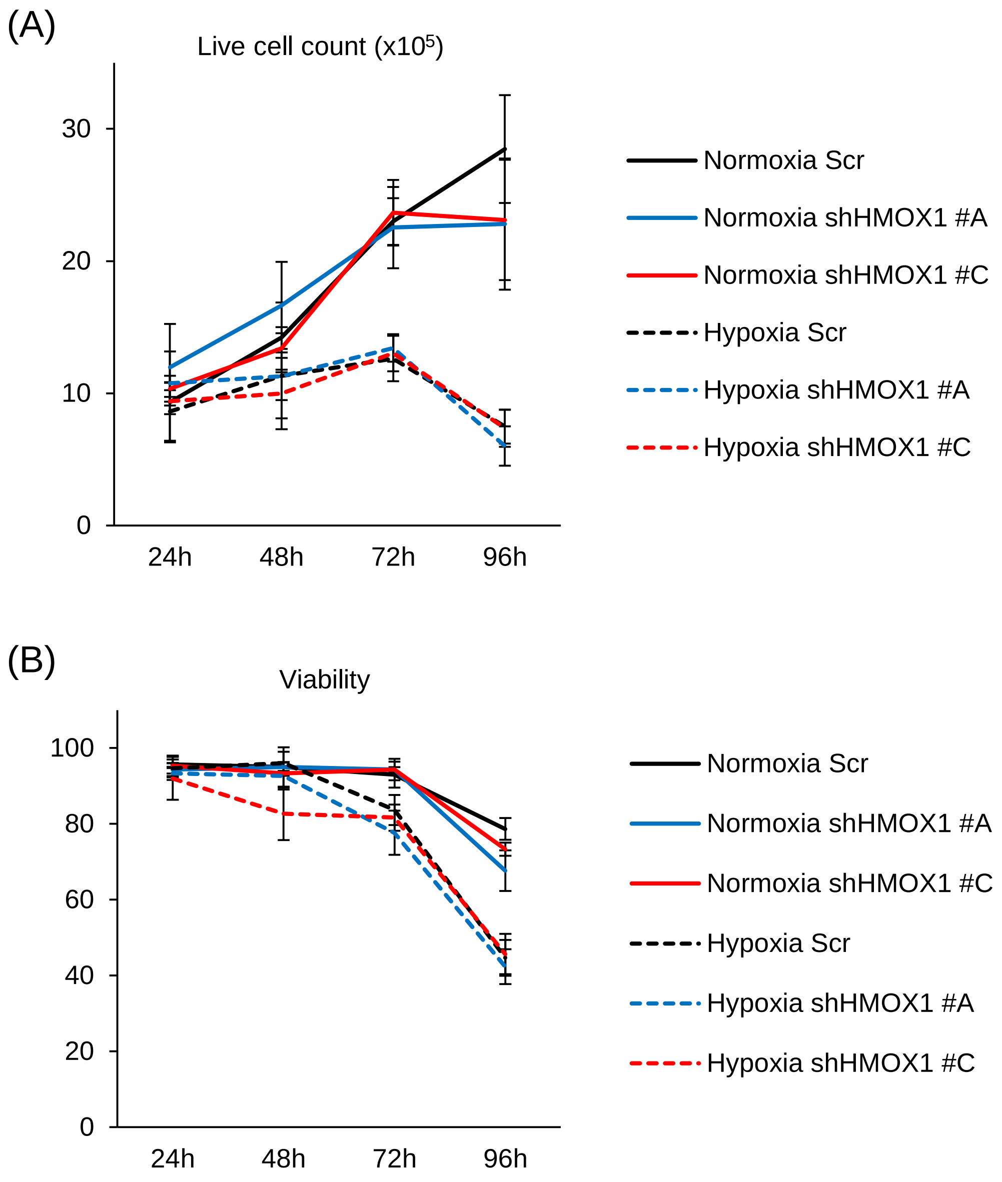

Figure S2. HMOX1 knockdown did not have an effect on the growth curve and viability in KMS-11 cells.
Cell counts and viability of KMS-11 cells stably transduced with shHMOX1 #A, #C, or control scrambled shRNA (Scr) were assessed using an auto cell counter and trypan blue.

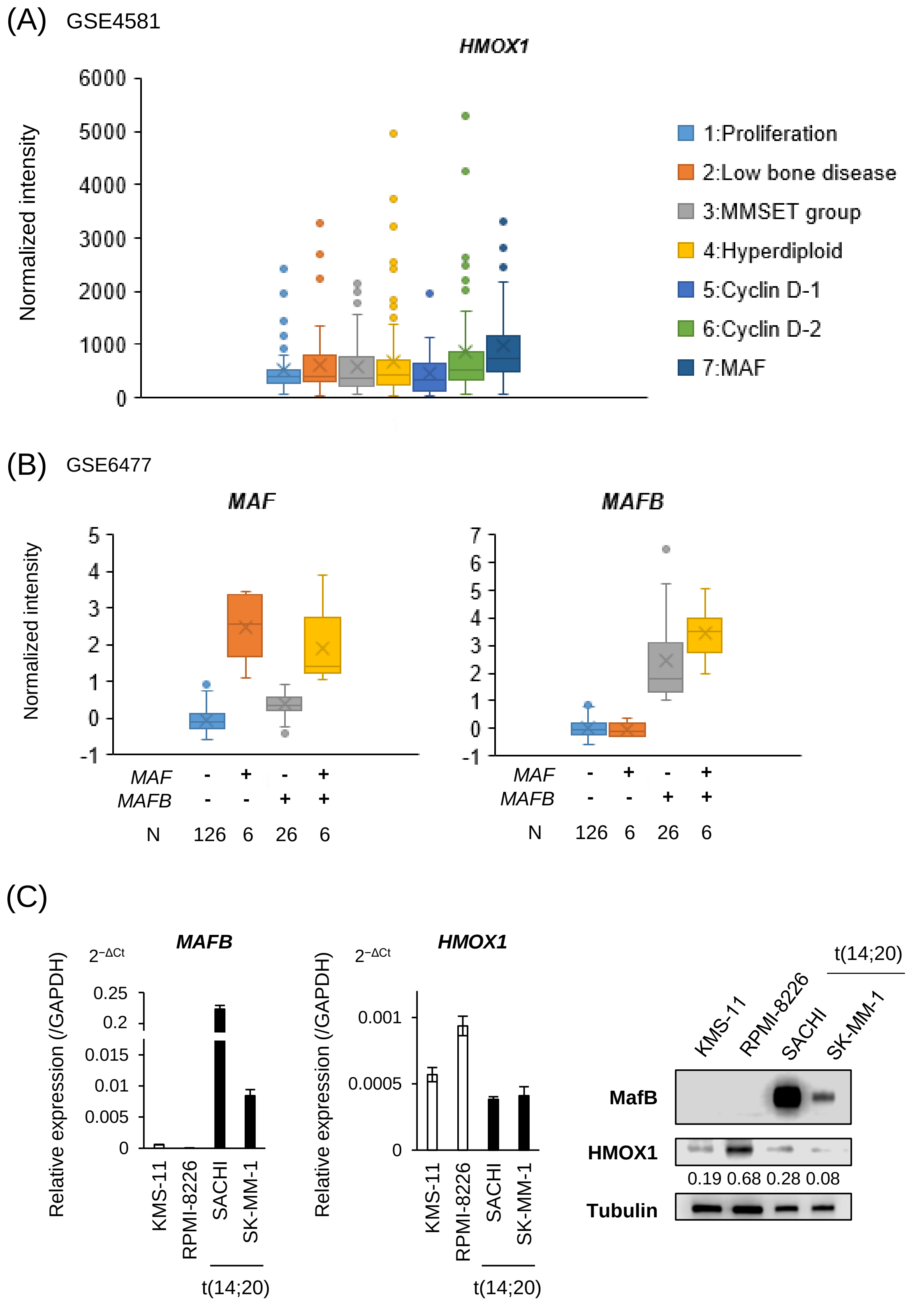

Figure S3. Relationship between *HMOX1* and *MAFB* in publicly available datasets.

(A) Expression of *HMOX1* in the seven indicated gene expression clusters.

(B) Patient samples in the dataset were classified by *MAF* and *MAFB* expression. The relative expression value used as the cutoff was $\log_2$ >1.0.

(C) The expression of MAFB and HMOX1 in indicated four myeloma cell lines. Left panel: qRT-PCR, Right panel: western blot.
